# Clinical overlap between functional neurological disorders and autism spectrum disorders: a preliminary study

**DOI:** 10.1007/s10072-022-06048-1

**Published:** 2022-05-05

**Authors:** Veronica Nisticò, Diana Goeta, Adriano Iacono, Roberta Tedesco, Barbara Giordano, Raffaella Faggioli, Alberto Priori, Orsola Gambini, Benedetta Demartini

**Affiliations:** 1 Dipartimento di Scienze della Salute, Università degli Studi di Milano, Presidio San Paolo, via A. di Rudinì, 8, 20142 Milano, Italy; 2 “Aldo Ravelli” Research Center for Neurotechnology and Experimental Brain Therapeutics, University of Milan, Milan, Italy; 3 Dipartimento di Psicologia, Università degli Studi di Milano Bicocca, Milan, Italy; 4 U.O. di Psichiatria, Presidio San Carlo, ASST Santi Paolo e Carlo, Milano, Italy; 5 U.O. di Psichiatria 52, Presidio San Paolo, ASST Santi Paolo e Carlo, Milano, Italy; 6 III Clinica Neurologica, Presidio San Paolo, ASST Santi Paolo e Carlo, Milano, Italy

**Keywords:** Functional Neurological Disorders, Functional Neurological Symptoms, Autism Spectrum Disorders, Autistic Traits, Sensory Perception, Bayesian Models

## Abstract

Functional neurological disorders (FNDs) and autism spectrum disorders (ASDs) share common features in terms of deficits in emotion regulation and recognition, sensory sensitivity, proprioception and interoception. Nevertheless, few studies have assessed their overlap. We recruited 21 patients with FNDs, 30 individuals with ASDs without intellectual disabilities and 45 neurotypical adults (NA). Participants completed: the Autism Quotient (AQ); the Ritvo Autism Asperger Diagnostic Scale-Revised (RAADS-R); and a questionnaire assessing functional neurological symptoms (FNS). ASDs participants also completed the Sensory Perception Quotient-Short Form (SPQ-SF35), assessing sensory sensitivity. In the FNDs sample, no patient scored above the clinical cut-off at the AQ and the 19% scored above the cut-off at the RAADS-R, a prevalence similar to the one we found in NA (15.6%; both *p* > 0.05). The 86.7% of participants with ASDs reported at least one FNS, a prevalence significantly higher than the NA one (35.6%, *p* < 0.001). In the ASDs sample, tactile hypersensitivity was found to be a risk factor for functional weakness (OR = 0.74, *p* = 0.033) and paraesthesia (OR = 0.753, *p* = 0.019). In conclusions, FNDs individuals did not present autistic traits more than NA, but ASDs individuals presented a higher number of FNSs than NA; this rate was associated with higher sensory sensitivity, especially in the touch domain.

**Supplementary Information:**

The online version contains supplementary material available at 10.1007/s10072-022-06048-1.

## Introduction

Functional neurological disorders (FNDs) and autism spectrum disorders (ASDs) are two common neuropsychiatric conditions, affecting childhood and adulthood. FNDs are characterized by the presence of neurological symptoms that cannot be explained by typical neurological diseases or other medical conditions, nevertheless determining clinically significant discomfort or impairment [1, 2]. ASDs include a wide variety of conditions, sharing the common core of “persistent deficits in social communication and social interaction across multiple contexts” [1], that can be thought as a continuum, ranging from a pole with severe delay in cognitive, social and emotional development, to a pole with selective impairment in understanding and responding to social cues, without intellectual disabilities. Albeit FNDs and ASDs are both quite common in the general population (with a prevalence of, respectively, 0.05% [3] and 1.1% [4]), their pathophysiological mechanisms are still poorly understood. Moreover, although FNDs and ASDs have always been considered two different entities, from a clinical and pathophysiological perspective they might share some common features. Previous studies showed that patients with FNDs and individuals with ASDs present deficits in emotion regulation and recognition, in terms of recognizing emotions of others and one’s own emotions (alexithymia) [5–7]. Moreover, both groups present deficits in interoception, defined as the process of perceiving one’s own internal state [8, 9]. In this scenario, it has been proposed that both individuals with FNDs and with ASDs struggle to translate interoceptive signals into higher-order brain representations [8, 10], suggesting that they have difficulties integrating their physiological responses to emotional cues into overt emotional judgements. Moreover, the difficulty to correctly perceive interoceptive signals might be linked to the phenomenon of sensory over-responsivity, a condition characterized by exaggerated or prolonged negative response to sensory stimuli. Sensory over-responsivity is known to be a key feature of ASDs [1], but it has been described also in patients with FNDs: Ranford and colleagues [11] recently showed that patients with FNDs commonly report that sensory experiences might trigger their functional neurological symptoms (FNS). Finally, both the disorders are often associated with other specific psychiatric symptoms, such as depression or anxiety, which may represent the final epiphenomenon, along with the disorder itself, of the pathophysiological mechanisms described above.

Despite this background, only two studies have previously assessed the comorbidity between FNDs and ASDs. Miyawaki et al. [12] reported the case of a 10-year-old girl with ASDs without intellectual disabilities who developed psychogenic non-epileptic seizures (PNES) while she was treated for benign childhood epilepsy. More recently, Mc Williams et al. [13] found that 16.9% of their sample of children and adolescents with PNES also presented a previous diagnosis of ASDs; nevertheless, this study was limited by the retrospective design. Moreover, no studies have ever assessed the prevalence of ASDs traits in patients with FNDs, nor investigated the presence of FNS in a sample of adult patients with ASDs without intellectual disabilities.

Aims of the present study were: (i) to assess the prevalence of autistic traits in a sample of adult patients with FNDs and (ii) to assess the prevalence of FNS in a sample of adult individuals with ASDs without intellectual disabilities; in this sample, we also evaluated the presence of a possible association between sensory sensitivity and FNS.

## Methods

### Participants

Twenty-one consecutive patients with FNDs and thirty consecutive individuals with ASDs without intellectual disabilities were recruited at the tertiary level neuropsychiatric outpatient clinic of San Paolo Hospital in Milan. Diagnosis of FNDs was made by a neurologist and a psychiatrist according to DSM-5 diagnostic criteria [1]. Individuals with ASDs were diagnosed by a psychiatrist and a psychologist according to DSM-5 criteria [1] and the Module 4 of the Autism Diagnostic Observation Schedule—2nd version (ADOS-2) [14]. Forty-five neurotypical adults (NA) with no psychiatric or neurological diagnosis were recruited amongst hospital staff and their acquaintances and served as a control group.

Exclusion criteria were: (i) age less than 18 years; (ii) inability to communicate with the researcher or complete questionnaires because of language difficulties, severe learning disabilities (I.Q.<70) or dementia; and (iii) any other serious neurological or medical illnesses.

The study was approved by the local ethics committee. All participants signed an online-written informed consent form.

### Materials and procedure

Through an online questionnaire, demographic and clinical information was collected. Subsequently each participant completed the following questionnaires: (i) the Autism Quotient (AQ) [15], a 50-item self-reported questionnaire measuring the degree to which an adult without intellectual disabilities presents autistic traits; (ii) the Ritvo Autism Asperger Diagnostic Scale-Revised (RAADS-R) [16], an 80-item validated instrument designed to assist clinicians diagnosing ASDs in adults; and (iii) an ad hoc questionnaire drawn up from the Edinburgh Neurosymptoms Questionnaire [17] assessing the presence of specific FNS (Supplementary Materials). Each question was independently evaluated by two psychiatrists, blind with respect to the patient’s diagnosis, who decided whether the symptomatology reported was suggestive of FNS. A continuous Total Score, ranging from 0 (absence of FNS) to 8 (presence of all the FNS assessed), was calculated by summing the number of FNS presented by each participant. Finally, participants with ASDs completed the Sensory Perception Quotient-Short Form (SPQ-SF35) [18], a 35-item self-reported questionnaire investigating hyper- or hyposensitivity in the 5 modes of perception. A Total Score was calculated so that the lower the score, the lower the sensory threshold (i.e. the higher the sensory sensitivity).

### Statistical analysis

Statistical analysis was conducted with SPSS v.26 (p<0.05 deemed significant). First, descriptive statistics were calculated for each group. Pearson’s Chi-square test was run to investigate whether: (i) the prevalence of participants scoring above the cut-off at the AQ and the RAADS-R was equally distributed between FNDs and NA groups; and (ii) the prevalence of participants presenting at least one FNS was equally distributed between ASDs and NA groups. Within the ASDs group, to assess the potential association between sensory perception and FNS, Pearson’s correlational analysis between the SPQ-SF35 Total Score and the FNS Questionnaire Total Score was run. To investigate whether hypersensitivity in specific sensory domains predicted the presence of each FNS, a series of stepwise binary logistic regressions were run within the ASDs group, with the SPQ-SF35 subscales as predictor and each FNS as dichotomic dependent variable.

## Results

Within the FNDs group, 4 subjects out of 21 were male. Mean age was 42.9 (SD =13.02). No subject scored above the cut-off at the AQ (mea*n* =15.76, SD = 5.41), while 4 subjects (19%) scored above the cut-off at the RAADS-R (mea*n* =44.29, SD = 32.41).

Within the ASDs group, 16 subjects out of 30 were male. Mean age was 39.67 (SD = 12.18). Twenty-six individuals with ASDs (86.7%) reported at least one FNS. A negative correlation emerged between the SPQ-SF35 Total Score and the number of FNS (r = - 0.381; *p* = 0.038), suggesting that the lower the sensory threshold is, the higher the number of FNS presented by ASDs participants is. A logistic regression analysis showed that, out of the five SPQ-SF35 subscales, only the subscale Touch was associated with the FNS functional weakness (OR = 0.74, 95% CI [0.561; 0.977], *p* = 0.033 at Model 1—percentage of correct prediction: 83.3%) and paraesthesia (OR = 0.753, 95% CI [0.594; 0.954], *p* = 0.019 at Model 1—percentage of correct prediction: 76.7%).The other FNS were not associated with any of the SPQ-SF35 subscales.

Within the NA group, 28 subjects out of 45 were male. Mean age was 35.36 (SD = 11.85). One participant (2.2%) scored above the cut-off at the AQ (mea*n* =13.47, SD = 6.66), while 7 subjects (15.6%) scored above the cut-off at the RAADS-R (mea*n* =38.09, SD = 32). Sixteen individuals (35.6%) reported at least one FNS; in particular, 7 presented one FNS and 9 presented two or more FNS. Distribution of the specific functional symptoms was as follows: 4 participants presented loss of consciousness; 1 weakness; 6 paraesthesia; 8 tremor; 4 chronic pain; and 8 chronic fatigue (Table 1)

**Table 1:** Demographic and clinical information for individuals with ASDs, patients with FNDs and NA

	ASDs (*N* =30)	FNDs (*N* =21)	NA (*N* =45)
Age, mean (SD)	39.7 (12.2)	42.9 (13)	35.4 (11.8)
Sex, M/F	16/14	4/17	28/17
WAIS-IV Verbal Comprehension Index	129.9 (15.9)	N/A	N/A
WAIS-IV Perceptual Reasoning Index	116.2 (14.4)	N/A	N/A
AQ, mean (SD)	37.8 (4.3)	15.8 (5.4)	13.5 (6.7)
AQ Clinical, N (%)	30 (100%)	0 (0%)	1 (2.2%)
RAADS-R, mean (SD)	156 (30.1)	44.3 (32.4)	38.1 (32)
RAADS-R Clinical, N (%)	30 (100%)	4 (19%)	7 (15.6%)
FNS Questionnaire, mean (SD)	2.1 (1.5)	3.8 (1.6)	0.7 (1.1)
no FNS, N (%)	4 (13.3%)	0 (0%)	29 (64.4%)
one or more FNS, N (%)	26 (86.7%)	21 (100%)	16 (35.6%)
Loss of consciousness, N (%)	5 (16.7%)	11 (52.4%)	4 (8.9%) 1 missing
Weakness, N (%)	6 (20%)	13 (61.9%)	1 (2.2%)
Paraesthesia, N (%)	15 (50%)	11 (52.4%)	6 (13.3%)
Cognitive disorders, N (%)	0 (0%)	6 (28.6%)	0 (0%) 3 missing
Tremor, N (%)	8 (26.7%)	12 (57.1%)	8 (17.8%)
Chronic pain, N (%)	7 (23.3%)	14 (66.7%)	4 (8.9%)
Chronic Fatigue, N (%)	21 (70%)	17 (82%)	8 (17.8%)
Stuttering, N (%)	0 (0%)	1 (4.8%)	0 (0%)
SPQ-SF35 Total Score, mean (SD)	42.7 (15.5)	N/A	N/A

Abbreviations: ASDs = Autism Spectrum Disorders group; AQ = Autism Quotient; Clinical = participants scoring above the clinical cut-off; F = female; FNDs = Functional Neurological Disorders group; FNS = Functional Neurological Symptom; M = male; NA = Neurotypical Adults; N/A = not applicable; RAADS-R = Ritvo Autism Asperger Diagnostic Scale-Revised; SD = Standard Deviation; SPQ-SF35 = Sensory Perception Quotient-Short Form; WAIS-IV: Wechsler Adult Intelligent Scale—IV edition.

**Fig. 1:**
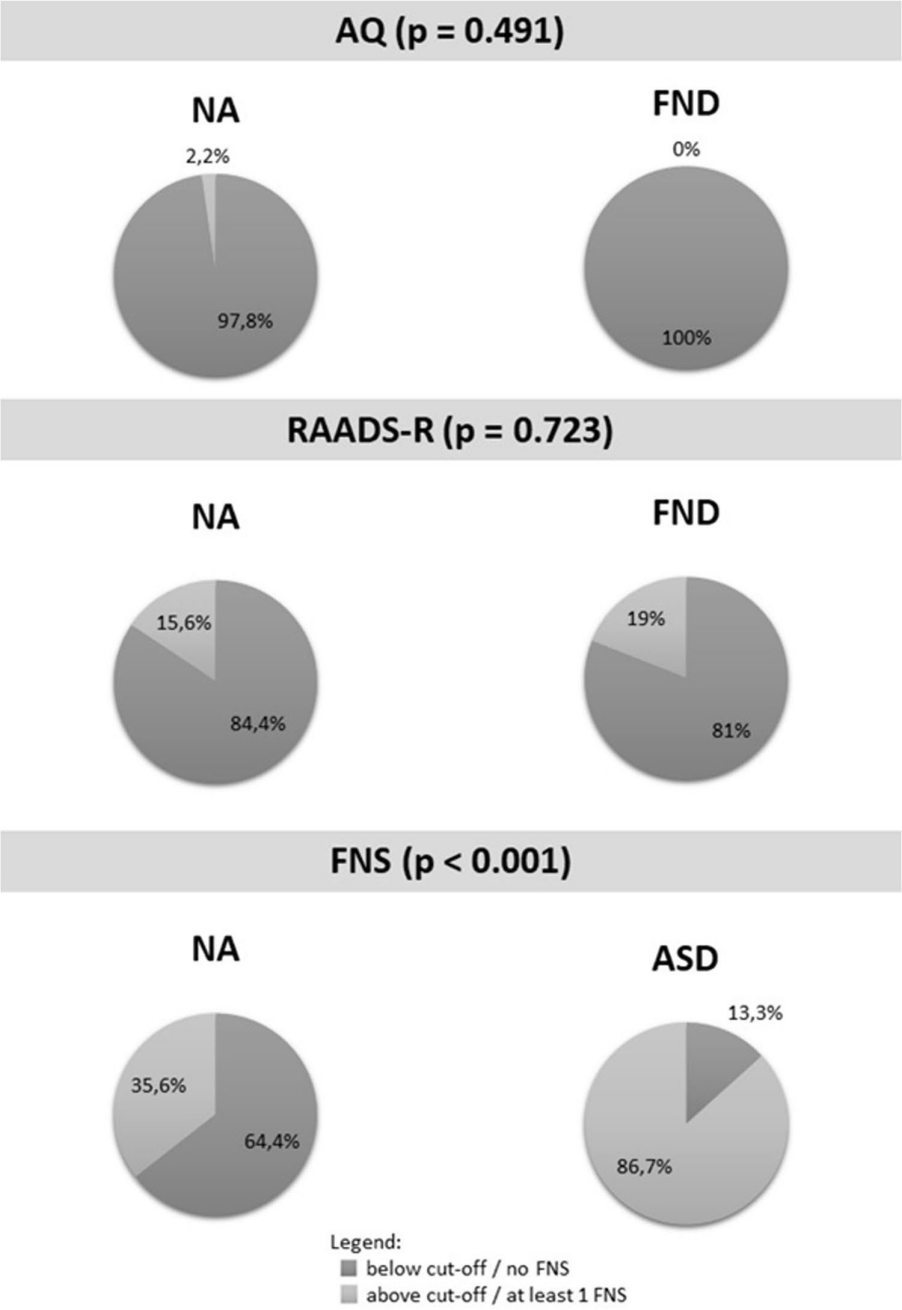
Prevalence of participants scoring above the cut-off at the AQ and the RAADS-R in FNDs and NA groups, and prevalence of participants presenting at least one functional symptoms in ASDs and NA groups. p values refers to c^2^ analysis. Abbreviations: ASDs = Autism Spectrum Disorders group; AQ = Autism Quotient; FNS = Functional Neurological Symptom; NA = Neurotypical Adults; RAADS-R = Ritvo Autism Asperger Diagnostic Scale-Revised; FNDs = Functional Neurological Disorders group

Chi-square analysis showed that: (i) there was no significant difference between FNDs and NA groups with respect to the number of participants scoring above the AQ (χ (1) = 0.474, *p* = 0.491) and the RAADS-R (χ (1) = 0.126, *p* = 0.723); and (ii) the number of participants presenting at least one FNS was significantly higher in ASD than in NA group (χ (1) = 19.084, *p* < 0.001) (Fig. 1).

## Discussion

The first aim of this study was to assess the prevalence of autistic traits in a population of adult patients with FNDs. Our results showed that, at the AQ, no patient with FNDs scored above the clinical cut-off of 32, while at the RAADS-R the 19% scored above the cut-off, a prevalence similar to the one we found in our sample of NA (15.6%). The second aim of the present study was to evaluate the presence of FNS in a population of adult patients with ASDs without intellectual disabilities. Here we showed that the 86.7% of our sample reported at least one FNS, a prevalence significantly higher than the one encountered in our sample of NA (35.6%).

A possible interpretation of these findings may be linked to the Bayesian model proposed by Edwards and colleagues [10]: Patients with FNDs, given their alexithymic traits, might fail to interpret correctly as anxiety the autonomic arousal occurring during a physical precipitating event, and interpret these sensations as symptoms of physical illness [5]; moreover, given their obsessive–compulsive personality traits [5] they keep on focusing their attention on their symptoms and checking them again and again; finally, given their reduced interoceptive ability [5, 9], they cannot successfully update their prior belief of sensations which results to be strict and poorly modifiable. In a Bayesian aetiological framework, in fact, strict prior beliefs or expectations and abnormal attention towards the body are thought to be key mechanisms of the pathophysiology of FNDs. The ultimate goal of the perception process, according to the Bayesian theory, is to accurately update expectations of sensation, the so-called prior beliefs, in order to keep the gap between them and the bottom-up afferences as low as possible. This gap is named prediction error [10]. Here, we might speculate that also individuals with ASDs given their well-known deficit in interoception, alexythimic traits [8] and over-responsivity to sensory input [1, 19] might be more susceptible to experience their autonomic arousal as physical symptoms without an effective and rapid update of prior expectations, thus leading to the emergence of FNS.

On logistic regression analysis, only the subscale “Touch” of SPQ-SF35 was associated with the significantly more frequent FNS in the ASDs group, namely functional weakness and paraesthesia; in other words, a lower threshold for the sense of touch, delivered from periphery to the central nervous system (CNS) by myelinated fibre A*β*, was found to be a risk factor for the development of functional weakness and paraesthesia.

We interpreted these data in light of the well-known low affective touch and pain threshold of ASD patients [20, 21]. Pain stimuli and affective touch are delivered from periphery to the central nervous system (CNS) by myelinated Aδ fibres and unmyelinated C fibres. According to “the gate control theory”, myelinated A*β* fibres inhibit Aδ/C fibres. The more the A*β* fibres are active the more they inhibit the Aδ/C ones, determining a modified and reduced sensory information up to the cortex. Bottom-up information therefore deviate from the original expectations which, as already mentioned, are very strict and poorly malleable, resulting in a bigger predictive error. In a Bayesian framework, in order to keep the prediction error as low as possible, we speculate that the brain “produces” functional paraesthesia to compensate the difference between bottom-up and top-down information. Regarding functional weakness, it is already established that a correct sensory afference is essential to a successful motor planning. In addition, ASDs patients usually manifest motor disturbances such as reduced grip strength, clumsiness, incoordination, showing a pre-existing overall weakness of the motor function (20). Here, according to the “gate control theory”, the over inhibition of Aδ/C fibres given by the over activation of A*β* fibres, could cause a reduced and quality modified sensory signal in the cortex, causing an incorrect motor planning, ultimately resulting in functional weakness.

A major limitation of our study is not having assessed sensory perception in our group of FNDs participants, and therefore, we cannot test whether the association between a lower sensory threshold and the emergence of FNS would be significant also in a sample with a diagnosed FNDs. Up to date, few studies assessed sensory perception in FNDs. Ranford and colleagues [11] found that sensory experiences in patients with FNDs were perceived at low neurological thresholds more frequently than the general population, and used poorer coping strategies for managing the emotional, behavioural and physiological responses to sensory experiences. On the other hand, Brown et al. [22] found that healthy subjects who scored higher on a questionnaire assessing conversion and dissociation experiences, had a more liberal response criterion in a signal detection task, attributable not to a greater sensitivity but to more false alarms, which, according to Edwards and colleagues [10], might be evidence of an abnormally weighted prior expectation, at the expense of the real sensory stimulation. Finally, Morgante et al. [23, 24] assessed with psychophysical techniques the processing of somatosensory and painful stimuli in patients diagnosed with Functional Dystonia (F-Dys), another FND phenotype. In a first study [23] they found increased temporal discrimination threshold (TDT) in F-Dys patients, compared to HC, suggesting an impaired processing of somatosensory input at the central level (since TDT of tactile stimuli relies upon several cortical and subcortical areas, including the somatosensory areas, the pre–supplementary motor area, the anterior cingulate cortex and the basal ganglia, as reported by the authors). In a second study [24], they examined: (i) tactile and pain thresholds, defined as the intensity at which sensations changed from unpainful to slightly painful; and (ii) pain tolerance, defined as the intensity at which painful sensations were considered intolerable. It is noteworthy that these two aspects of pain can be selectively tested, and they, respectively, account for two neuroanatomical components of the so-called pain matrix: the lateral pathway, projecting to the lateral thalamus and then to primary and secondary somatosensory areas (sensory-discriminative component of pain), and the medial pathway, projecting to the medial thalamic nuclei and limbic structures, such as the anterior cingulate cortex and the insular cortex (affective–cognitive component of pain). The authors found a significantly enhanced pain tolerance in all body regions in patients with F-Dys compared to a group of patients with idiopathic cervical dystonia and to a group of healthy controls, suggesting an abnormal functioning of the cognitive emotional components of pain (i.e. the medial pathway) in FND patients; according to the authors, their result is in line with several previous findings suggesting an alteration in the connectivity between the motor areas and limbic systems in FND (for a review see [25]). On the contrary, the authors found no differences in tactile and pain thresholds between patients with F-Dys and the control groups, suggesting that the sensory-discriminative component of pain would not be altered in this group of patients; this finding is apparently in contrast with our hypothesis that an altered sensory threshold might have a role in the production of functional symptoms; however, Morgante et al. [24] acknowledge that, in a Bayesian framework, the cognitive appraisal of pain tolerance can be influenced by the excessive attention directed towards the body and by top-down influences such prior beliefs, that would tend to modify any bottom-up sensory information. Overall, these studies point in the direction of an alteration of sensory and pain perception in FND, which deserves further investigation.

Other limitations of our study are: (i) all data were self-reported; (ii) up to date, there are no data available about the reliability of the FNS questionnaire implemented in the present study; moreover, given the fact that some FNS are investigated through only one single question [17], one might think that this questionnaire tends to overestimate the presence of FNS. On the one hand, comparing the results of our questionnaire to the clinical notes of previously diagnosed FNDs participants, the questionnaire appeared reliable in detecting symptoms suggestive of FNDs; on the other hand, it must be stated that the questionnaire did not convey a formal diagnosis of FNDs, which can be achieved only by identifying specific positive features at clinical examination [26]; hence, the questionnaire implemented here cannot be considered exhaustive and future studies should further investigate our preliminary findings through formal clinical examination; (iii) the absence of cognitive deficit and the exclusion of ASDs diagnosis were established in the FNDs and NA groups by means of clinical interview only; (iv) the numerosity of the groups are not balanced; moreover, although we excluded the presence of cognitive deficit in each group, we did not match the groups for educational level.

## Conclusions

In conclusion, our study gives preliminary evidence that individuals with ASDs without intellectual disabilities are more likely to experience FNS than the general population. This should be taken into account in everyday clinical practice. Given our results, we suggest to routinely screen ASDs patients for FNS and to administer them the SPQ-SF35 in order to identify those at higher risk to develop FNS.

## Supplementary Information

ESM 1 (DOCX 29 kb)
