# Supplementary material for: Clinical overlap between functional neurological disorders and autism spectrum disorders: a preliminary study
Source: Neurol Sci. 2022 May 5;43(8):5067–73. doi: 10.1007/s10072-022-06048-1 (PMC9349073; doi:10.1007/s10072-022-06048-1)
Supplement: Supplementary file 1 — (DOCX 29 kb) [file 10072_2022_6048_MOESM1_ESM.docx]

**Supplementary Materials**

**FNS Questionnaire – Italian Version**

| 1. Le capita o le è capitato di perdere coscienza? | Sì | No |
| --- | --- | --- |
| Se ha risposto Sì alla domanda precedente: | | |
| Cosa le succede mentre è incosciente? | Trema | |
|  | Rimane immobile | |
|  | Ha convulsioni | |
|  | Si irrigidisce | |
|  | Parla o fa cose di cui non si ricorda | |
| Ha avuto due o più episodi della durata superiore a dieci minuti? | Sì | No |
| Ha mai avuto rilascio sfinterico (perdita di feci/urine) durante un episodio? | Sì | No |
| si è mai morso involontariamente la punta della lingua, non i margini laterali, durante un episodio? | Sì | No |
| Al termine dell’episodio le capita di piangere o di sentirsi particolarmente irritabile/arrabbiato? | Sì | No |
| Durante l’episodio riesce a sentire le persone intorno a lei che la chiamano/le pongono domande ma lei non è in grado di rispondere? | Sì | No |
| Prima di un episodio, le capita di sentirsi distaccato dalla realtà che la circonda, di non avere completamente il controllo sul proprio corpo, di non avere completamente il controllo ciò che sta dicendo? | Sì | No |
| PER IL CLINICO: Nel complesso, le risposte a questo gruppo di domande sono suggestive di FNS? | Sì | No |
|  | | |
| 1. Soffre di debolezza muscolare a carico di uno o più parti del corpo? | **Sì** | **No** |
| Se ha risposto Sì alla domanda precedente: | | |
| Se la debolezza è a carico degli arti superiori, le capita frequentemente di far cadere gli oggetti che ha in mano? | Sì | No |
| La gravità della debolezza varia nell'arco della giornata e/o della settimana? | Sì | No |
| Le capita che la debolezza peggiori quando cerca di concertarsi sul movimento dell'arto affetto? | Sì | No |
| Le capita al contrario che quando è distratto la forza aumenti e globalmente la riuscita del movimento migliori? | Sì | No |
| Ha l’impressione che l’arto in questione non faccia parte di lei? | Sì | No |
| PER IL CLINICO: Nel complesso, le risposte a questo gruppo di domande sono suggestive di FNS? | Sì | No |
|  | | |
| 1. Le capita di avvertire parestesie (formicolii), o riduzione della sensibilità, che le danno la sensazione di avere il corpo diviso in due? | **Sì** | **No** |
| PER IL CLINICO: Nel complesso, le risposte a questo gruppo di domande sono suggestive di FNS? | Sì | No |
|  | | |
| 1. Soffre di disturbi di memoria? | **Sì** | **No** |
| Se ha risposto Sì alla domanda precedente: | | |
| Qual è l'ambito della sua vita che ha maggiormente subito l’impatto del deficit di memoria in oggetto? | Lavoro | |
|  | Partner | |
|  | Famiglia | |
|  | Altro: __________ | |
| Esiste un ambito della sua vita dove il disturbo di memoria non si presenta? | __________ | |
| Ha eseguito dei test di memoria prescritti da un medico, risultati nella norma? | Sì | No |
| PER IL CLINICO: Nel complesso, le risposte a questo gruppo di domande sono suggestive di FNS? | Sì | No |
|  | | |
| 1. Le capita di avere tremore o movimenti involontari a carico di una o più parti del corpo? | **Sì** | **No** |
| Se ha risposto Sì alla domanda precedente: | | |
| Il movimento in questione ha un inizio improvviso? | Sì | No |
| si è manifestato a seguito di un infortunio? | Sì | No |
| Può interrompersi improvvisamente e ricomparire dopo diverse ore? | Sì | No |
| Può scomparire del tutto se è impegnato in altre attività o se è distratto? | Sì | No |
| PER IL CLINICO: Nel complesso, le risposte a questo gruppo di domande sono suggestive di FNS? | Sì | No |
|  | | |
| 1. Soffre di dolori diffusi con frequenza quotidiana? | **Sì** | **No** |
| Se ha risposto Sì alla domanda precedente: | | |
| L'intensità del dolore cambia di giorno in giorno in diverse parti del corpo? | Sì | No |
| PER IL CLINICO: Nel complesso, le risposte a questo gruppo di domande sono suggestive di FNS? | Sì | No |
|  | | |
| 1. Si sente particolarmente stanco o privo di energie senza che le sue attività quotidiane siano cambiate rispetto a prima? | **Sì** | **No** |
| PER IL CLINICO: Nel complesso, le risposte a questo gruppo di domande sono suggestive di FNS? | Sì | No |
|  | | |
| 1. Soffre di balbuzie, il cui esordio è stato successivo a quando aveva 16 anni? | **Sì** | **No** |
| PER IL CLINICO: Nel complesso, le risposte a questo gruppo di domande sono suggestive di FNS? | Sì | No |

**FNS Questionnaire – English Version**

| 1. Have you been bothered by blackouts/loss of consciousness? | Yes | No |
| --- | --- | --- |
| If you answered “Yes” to the previous question: | | |
| What does it happen to you when you are unconscious? | You shake | |
|  | You stay/lie still | |
|  | You have convulsions | |
|  | You freeze | |
|  | You speak and/or do things you do not remember | |
| Have you had more than two episodes lasting more than 10 minutes? | Yes | No |
| Have you ever lost stools or urine during an episode? | Yes | No |
| Have you ever involuntarily bit the tip of your tongue, not the side edges, during an episode? | Yes | No |
| At the end of the episode, do you cry or do you feel particularly irritable/angry? | Yes | No |
| During the episode, can you hear the people around you calling / asking questions but you are unable to answer? | Yes | No |
| Before an episode, do you feel detached from the reality that surrounds you and/or do you feel not completely in control of your body/of what you are saying? | Yes | No |
| FOR THE CLINICIAN ONLY: Overall, are the answers to this group of questions suggestive of FNS? | Yes | No |
|  | | |
| 1. Do you suffer from muscle weakness affecting one or more parts of the body? | **Yes** | **No** |
| If you answered “Yes” to the previous question: | | |
| If the weakness is affecting the upper limbs, do you frequently drop the objects you are holding? | Yes | No |
| Does your limb weakness get worse or better at different times of the day? | Yes | No |
| Does concentrating on trying to move make the limb weakness worse? | Yes | No |
| When you are distracted does your strength increase and overall the success of the movement improve? | Yes | No |
| Does your weak limb feel like it does not fully belong to you? | Yes | No |
| FOR THE CLINICIAN ONLY: Overall, are the answers to this group of questions suggestive of FNS? | Yes | No |
|  | | |
| 1. Do you have numbness (tingling), or reduced sensitivity, that makes you feel like your body is cut in half? | **Yes** | **No** |
| FOR THE CLINICIAN ONLY: Overall, are the answers to this group of questions suggestive of FNS? | Yes | No |
|  | | |
| 1. Do you suffer from memory problems? | **Yes** | **No** |
| If you answered “Yes” to the previous question: | | |
| What is the area of ​​your life that has most suffered the impact of this memory deficit? | Job | |
|  | Partner | |
|  | Family | |
|  | Other: | |
| Is there an area in your life where the memory deficit does not show up? | __________ | |
| Have you performed any memory tests (prescribed by a doctor), which came out to be completely normal? | Yes | No |
| FOR THE CLINICIAN ONLY: Overall, are the answers to this group of questions suggestive of FNS? | Yes | No |
|  | | |
| 1. Have you been bothered by tremor or involuntary (an abnormal) movements affecting one or more parts of the body? | **Yes** | **No** |
| If you answered “Yes” to the previous question: | | |
| Did your tremor or abnormal movement start suddenly? | Yes | No |
| Did your tremor or abnormal movement start after an injury or accident? | Yes | No |
| Can it suddenly stop and reappear after several hours? | Yes | No |
| Does your tremor or abnormal movement ever stop when you are distracted or concentrating on something else? | Yes | No |
| FOR THE CLINICIAN ONLY: Overall, are the answers to this group of questions suggestive of FNS? | Yes | No |
|  | | |
| 1. Do you suffer from widespread pain, on a daily basis? | **Yes** | **No** |
| If you answered “Yes” to the previous question: | | |
| Is your pain worst in different parts of your body on different days? | Yes | No |
| FOR THE CLINICIAN ONLY: Overall, are the answers to this group of questions suggestive of FNS? | Yes | No |
|  | | |
| 1. Have you been lacking energy even if your daily activities did not change from before? | **Yes** | **No** |
| FOR THE CLINICIAN ONLY: Overall, are the answers to this group of questions suggestive of FNS? | Yes | No |
|  | | |
| 1. Do you have a stutter which started after you were more than 16 years old? | **Yes** | **No** |
| FOR THE CLINICIAN ONLY: Overall, are the answers to this group of questions suggestive of FNS? | Yes | No |
